# Analysis of Stochastic Strategies in Bacterial Competence: A Master Equation Approach

**DOI:** 10.1371/journal.pcbi.1000985

**Published:** 2010-11-11

**Authors:** Sandra H. Dandach, Mustafa Khammash

**Affiliations:** Department of Mechanical and Industrial Engineering, University of California, Santa Barbara, Santa Barbara, California, United States of America; University of Illinois at Urbana-Champaign, United States of America

## Abstract

Competence is a transiently differentiated state that certain bacterial cells reach when faced with a stressful environment. Entrance into competence can be attributed to the excitability of the dynamics governing the genetic circuit that regulates this cellular behavior. Like many biological behaviors, entrance into competence is a stochastic event. In this case cellular noise is responsible for driving the cell from a vegetative state into competence and back. In this work we present a novel numerical method for the analysis of stochastic biochemical events and use it to study the excitable dynamics responsible for competence in *Bacillus subtilis*. Starting with a Finite State Projection (FSP) solution of the chemical master equation (CME), we develop efficient numerical tools for accurately computing competence probability. Additionally, we propose a new approach for the sensitivity analysis of stochastic events and utilize it to elucidate the robustness properties of the competence regulatory genetic circuit. We also propose and implement a numerical method to calculate the expected time it takes a cell to return from competence. Although this study is focused on an example of cell-differentiation in *Bacillus subtilis*, our approach can be applied to a wide range of stochastic phenomena in biological systems.

## Author Summary

When exposed to stress, organisms react by taking actions that help them protect their DNA. ComK protein is a key regulator which activates hundreds of genes, including the genes encoding the DNA-uptake and recombination systems. In *Bacillus subtilis*, stress in the environment activates a sequence of chemical reactions that, driven by cellular noise, stochastically increases the level of ComK in some bacterial cells driving them from their original vegetative state into a competent state. Entrance into and exit from competence are stochastic switching events that the cell undergoes. In this work, we present a novel numerical method that allows the analysis of stochastic events in biological systems. We illustrate our method by computing the probability with which *Bacillus subtilis* enters in competence. We also present a method to analyze the sensitivity of stochastic events. We use this method to study the sensitivity of the probability of entrance in competence with respect to various gene expressions and degradation rates. We finally present a numerical method to calculate the expected time it takes a cell to return from competence. Although we studied the competence regulatory genetic circuit, our approach can be applied to a variety of stochastic events in biological systems.

## Introduction

Competence is the ability of a cell, usually a bacterium, to bind and internalize transforming exogenous DNA. Under stressful environments, such as nutrient limitations, some cells enter competence while other cells commit irreversibly to sporulation. Entry in competence is a transient probabilistic event that facilitates copying of the exogenous DNA [1], [2]. It has been shown that among a group of cells only a randomly chosen fraction enters in competence [3], [4]. Proper modeling and correctly accounting for noise in the model of this phenomenon is crucial to understanding the underlying biological explanation. The few cells that enter competence express a high concentration of the key regulator ComK, which activates hundreds of genes, including the genes encoding the DNA-uptake and recombination systems [5]–[7]. Competence is understood as a bistability pattern [4], [8] and the nonlinear system describing the competence regulatory circuit is an excitable dynamical system.

Auto-activation of the regulator ComK is responsible for the bistable response in competence development. Auto-activation of ComK, is essential and can be sufficient to generate a bistable expression pattern [9]–[11]. Specifically, the concentration of an inducer must cross a certain threshold to start the positive feedback. Different experimental studies concluded that an auto activation of ComK is the only needed factor for bistability to occur in the expression of this protein [9], [11], [12]. In [9], Smits et. al discuss the factors that determine the required threshold for the activation of ComK and deduce that other transcription factors can raise or lower the threshold. Although many proteins are involved in the regulation of competence, there are two main proteins that play a major role. Süel et al. [13] propose a deterministic model driven by an additive noise to describe the dynamics of competence regulation. We use the reduced order Stochastic Differential Equation model (SDE) presented in [13] to develop a discrete stochastic model for competence. Calculating the probability and the expected time for entering and returning from competence, requires solving for the splitting probabilities and the first moment passage time. The problem of calculating the first passage time has been studied heavily in the literature for the stochastic difference equations, Fokker Planck equations and some special cases of the CME (separable kernels or single specie). For a detailed treatment of this topic see [14]–[17] and references therein. Researchers usually use Monte-Carlo simulations to calculate the distribution of the first passage time when working with he CME (e.g. see [18] and references therein). We propose in this work, an alternative approach that makes it possible to calculate the states in which the system will be as time evolves. The main idea here is to aggregate regions of the state space over which specie evolve into absorbing states. This technique is useful in analytically computing the distribution of the first passage time, by providing a way to deal with the infinite dimension of the state space over which the system evolves.

The contributions of this paper are threefold. First, it provides a new method to calculate exact probabilities of biological phenomena where transient behaviors such as competence, which is the topic we chose to study here, occur. Second, it shows how to calculate sensitivities of the probabilities of passing to the transient state with respect to the system's parameters. Third, it gives a methodology to calculate the expected time that it takes a cell to return from its transient state. All these methods can be used to analyze any biological system that has the characteristic of switching between two states, while staying for a while in the unstable state.

In this paper we start by describing the chemical reactions and the deterministic model. We then generate the Chemical Master Equation (CME) of our proposed discrete stochastic model. The CME characterizes the evolution of the probability density of the different discrete states. We simulate it using the Stochastic Simulation Algorithm (SSA) and show how the solution can be approximated using the Finite State Projection method (FSP). We then conduct a sensitivity analysis studying the effect that the various system parameters have on the probability with which a cell enters in competence. This analysis shows the usefulness of our proposed numerical method in analyzing the roles of the different affinity, transcription and degradation rates, etc., in driving the cellular switching (between competence and vegetative states in this case). Finally, we analyze the roles of these parameters in determining the expected time a cell stays in competence.

## Materials and Methods

We introduce at the beginning the modeling techniques used to propose a set of equations that capture the behavior of interest. We then present our discrete stochastic Chemical Master Equation (CME) model, followed by the Stochastic Simulation Algorithm (SSA) used to approximate the solution of the CME. We proceed to present our Finite State Projection based method that makes it possible to analyze the CME exactly. We show how such a method can be tailored to answer many questions of biological interest.

### Deterministic model and chemical reactions

Competence is a physiological state that enables cells to bind and internalize transforming DNA. This state is accompanied by blockage of the essential cell's functions, and since this state is driven by the transcriptional factor ComK, it is no surprise that ComK synthesis is subject to a number of finely tuned regulatory circuits [19]. The gene regulatory model for competence has been presented and described in [13]. Entrance of a cell in competence is controlled by a set of molecular interactions. Initially ComK and ComS are present in the cell at basal levels. The transcriptional factor ComK activates its own expression through positive feedback. The MecA complex is a multiprotein assembly that includes the ClpP-ClpC proteases. Bound to MecA, ComK is degraded under the action of the ClpP-ClpC proteases. In stressful environments, the level of ComS is high and that favors entrance into competence since ComS competes with ComK to bind to MecA. Inhibition of the binding of ComK to MecA by competitive binding with MecA-ComS allows a higher number of free ComK molecules to be present, which finally triggers the positive feedback that further raises the number of ComK molecules driving the cell in competence. This rise in the number of ComK is specific to competence. Once the number of ComK molecules reaches a certain level, it acts as an inhibitor for ComS through repression. The increase in the level of ComK will also favor the binding of MecA-ComK complex which degrades ComK through the ClpP-ClpC proteases, starting the return from competence. At this point ComS is below its basal level because of the aforementioned repression from ComK. The level of ComK starts to decrease by degradation. The degradation has two effects: (1) the decrease in the level of ComK will affect the transcriptional auto regulatory positive feedback loop of ComK, and (2) the absence of ComK in high levels, favors the synthesis of ComS by releasing the ComK-mediated ComS repression. This continues until the cell eventually exits the state of competence. The above mentioned molecular interactions are described [13] by the following chemical reactions:
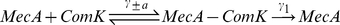


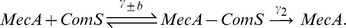
The rate equations describing the dynamics of the molecular reactions between the 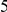 species model are the following:
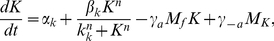
(1)

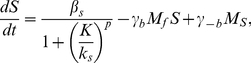
(2)

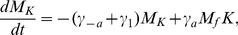
(3)

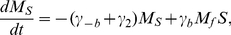
(4)where K, S, 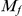, 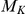 and 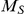 are the concentrations of ComK,ComS, MecA, MecA-ComK and MecA-ComS respectively. We give in Table 1 the values and the description of each of the parameters in Eq. 1–4.

**Table 1:** Parameter values as given in the literature.

Parameter values		
Parameter	Description	Value
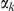	Basal expression rate of ComK	0.0028 nM/s
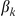	Saturating expression rate of ComK positive feedback	0.049 nM/s
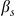	Unrepressed expression rate of ComS	0.057 nM/s
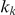	ComK concentration for half-maximal ComK activation	100 nM
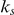	ComK concentration for half-maximal ComS repression	110 nM
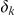	Unrepressed degradation rate of ComK	0.0014 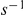
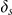	Unrepressed degradation rate of ComS	0.0014 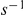
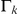	ComK concentration for half-maximal degradation	500 nM
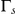	ComS concentration for half-maximal degradation	50 nM
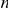	Hill coefficient of ComK positive feedback	2
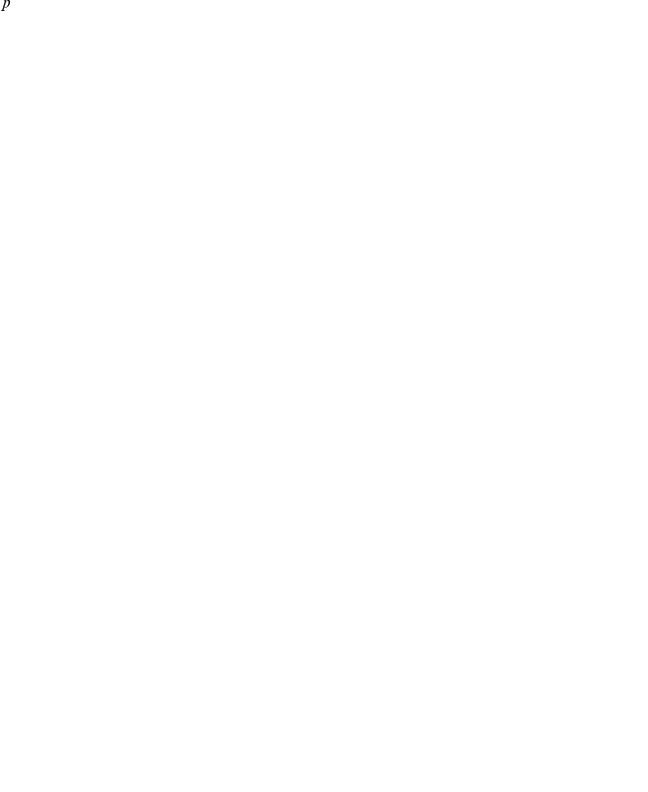	Hill coefficient of ComS repression by ComK	5

If one further assumes that the reactions of degradation of 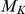 and 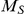 are much faster than the other reactions, 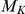 and 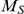 can then be eliminated through time scale separation [13], [20] and the conservation law:
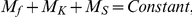
giving the following reduced model for the dynamics of competence:
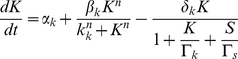
(5)

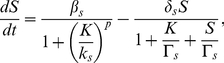
(6)where
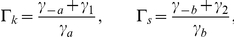
and
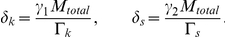



In their paper Süel et al. [13] analyze the excitable dynamical system described above. They present a phase diagram where they study the nullclines and the vector field of the dynamical system. Their analysis gives insight about the vegetative and competent states analyzed in this work. As we already stated, under the same conditions some cells enter into competence while other cells do not. Entry in competence is a random event, and in order to properly model the cell's behavior, we need to include the effect of noise on the dynamics of competence. In their analysis Süel et al. [13] account for stochasticity by adding white gaussian noise terms in Eq. 6. This drives the excitable dynamical system presented in Eq. 5–6 into long excursions when the noise magnitude is large enough. These long excursions correspond to a high level of ComK indicating entry into a state of competence. The problem with this approach is that reaching a competent state is highly dependent on the magnitude of the additive noise. The dynamics of the system in Eq. 5–6 are such that if the initial number of molecules of ComK and ComS is in the neighborhood of the fixed point of the dynamical system described in Eq. 5–6, the number of molecules for both species will stay in the vicinity of that point without taking long excursions. If on the other hand, the number of molecules is driven beyond a threshold, the dynamical system in Eq. 5–6 will have a totally different behavior. The number of molecules of ComK will increase significantly because of ComK auto-activation through positive feedback; In other words, the cells will enter in competence. Here we would like to analyze the stochastic behavior of the dynamics of the competence regulatory circuit taking into account the internal noise in the environment of the cell without having a direct control on the magnitude of the noise driving the regulatory circuit. To do so, we model the stochasticity in the chemical reactions using the CME. We look at the problem at the molecular level and propose four reactions to model the system in Eq. 5–6. The four reactions are:
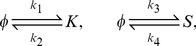
(7)with the following reaction rates:
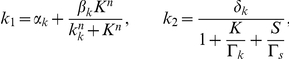


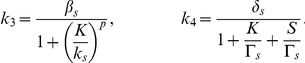
These reactions will serve as the starting point for developing and simulating a discrete stochastic model for competence in the next section.

### Discrete stochastic model and analysis methods

In order to compute the probability of entering into competence we use the CME to describe the stochastic chemical kinetics. Once we derive the CME, we simulate it using the Monte-Carlo based SSA. We then use the FSP method to obtain a finite dimensional solution to the infinite dimensional CME. In the CME, the state vectors indicate the number of molecules of each of the two species of interest: ComK and ComS. The CME describes the evolution of the probability that the number of molecules of each of the species has a given value. The dynamics of the evolution of the probability density vector are directly related to the chemical reactions. Starting from a number of molecules 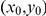, the probability of being at 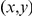 molecules at time 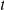 has the following dynamics:
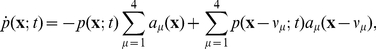
(8)where 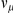 is the propensity vector and it represents the change that reaction 
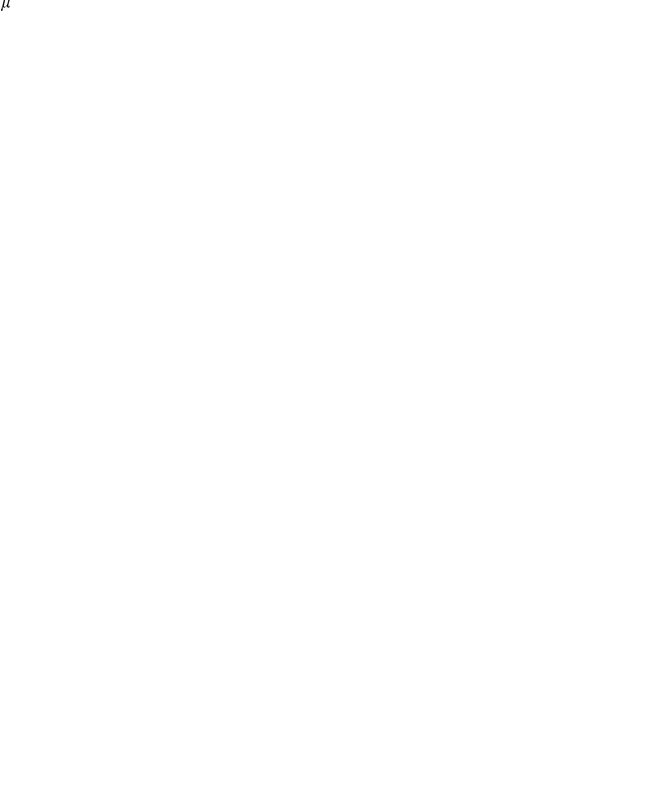
 will have on the number of molecules of each of the species. For example reaction 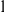 increases ComK by one molecule and leaves the number of molecules of ComS unchanged so the propensity vector 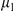 is 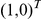, 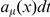 denotes the probability that the reaction 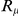 will occur in the next infinitesimal time interval 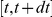. Written in vector form, the CME becomes
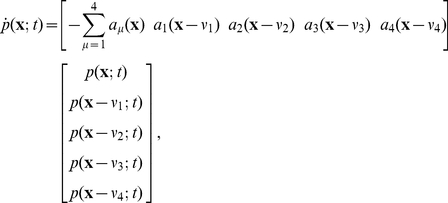
(9)where 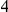 corresponds to the number of reactions that the species would go through. Let 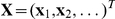 be a vector of the possible states of the system. Let 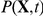 be the corresponding vector of probabilities of the states in 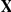 computed at time 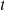. 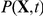 evolves according to the equation
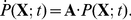
(10)In general, 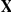 may be infinite, resulting in an infinite dimensional system.

### Stochastic Simulation Algorithm

Getting the exact value for the solution to the CME is not generally an easy task. In this part we introduce the SSA that is normally used to simulate Eq. 9. The SSA is a Monte-Carlo based algorithm that generates sample paths for the underlying stochastic process. Gillespie introduced this algorithm in 1977 [21]. Reactions are modeled as a random event whose occurrence depends in a non linear manner on the number of molecules through the reaction rates. The algorithm can be summarized as follows:

1. **Initialization:** Initialize the number of molecules in the system as well as the reaction rates.
2. **Reaction:** Generate random numbers that will correspond to a choice of a reaction. The probability of a reaction being chosen is proportional to the number of molecules involved in it.
3. **Number of molecules:** Update the number of molecules that were involved in the reaction.
4. **Time:** Update the time by the reaction time and repeat.

What we described above is a a basic summary of the algorithm, interested readers are referred to [21] for more details.

### Finite State Projection

**Figure 1:**
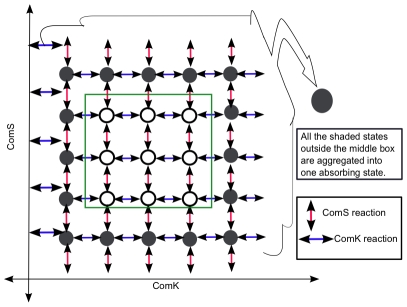
Projection of infinite lattice into a finite subspace. The probability density vector evolves on an infinite integer lattice as shown by the arrows. A boundary region of interest is chosen (shown as a *box* in the figure). In this region all the reactions are maintained. Outside the region all the states are aggregated into one absorbing state, and the reactions leaving the region are maintained, while return from the outside to the inside of the region is prohibited by deletion of the reactions. We chose the maximum value of 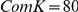, so that we detect the probability of leaving this boundary region within the reactions run time we are interested in.

The CME derived in Eq. 9 describes the evolution of the probability density vector of the number of molecules. Using SSA to get an estimate of the probability of entering into competence is easy to implement. However, a large number of simulations is required for a reasonably accurate estimate to be obtained. Aside from being time consuming, the algorithm has the drawback of lacking an accurate bound on the estimation error. In addition, analyzing the effect that different parameters have on the probability with which a cell enters in competence, requires the repetition of a large number of SSA simulations while changing those parameters of interest. This is numerically very costly. An alternative method in dealing with the CME is to compute an analytical expression for the probability of being in each state. The FSP method introduced in [22] provides a way to compute these probabilities. The probability density vector described in Eq. 9 allows molecules to evolve on an infinite lattice (Fig. 1) and therefore gives an infinite dimensional system. The idea behind FSP is to choose a suitable subset of the lattice in which one retains all the states and chemical reactions (transitions) found in the original system, while aggregating the remaining states in the lattice into one absorbing state. Transitions that drive the states outside the region are retained, while those that allow return to the selected finite region are deleted (see Fig. 1 for illustration). The finite state projection method gives the probability of being at any of the states inside the specified region at any point in time [22]. In this problem we are interested in finding the probability with which the pair (ComK,ComS) enters a region 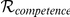 corresponding to the cell entering a state of competence. The sum of the probabilities of a cell being in 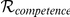 and of the probability of being anywhere else in the state space has to equal one at all times. Moreover, if we divide the state space of the two proteins ComK and ComS in two regions, then the probability of the cell being inside the first region without ever leaving it, and the probability of leaving the first region once within a time 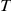 should sum to one. These properties make FSP a very well suited numerical method to solve our problem.

In the finite model all the states outside the projection region are aggregated into one absorbing state: 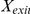 (see Fig. 1 for an illustration). The probability vector at time 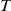 is given as in Eq. 10 by
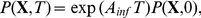
(11)where 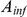 is an infinite matrix and 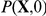 is the initial distribution of the probabilities, that is a vector with infinite entries, where each entry corresponds to a probability with which the system starts with a given number of molecules. Using FSP we can project the infinite system in Eq. 11 into the following finite system:
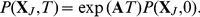
(12)In this case, **A** becomes a finite matrix, and 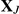 is the finite vector of projected states. We build the finite matrix 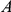 as follows
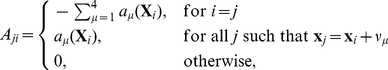
where 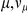 and 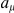 are the terms appearing in Eq. 8. If 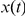 denotes the underlying stochastic process, 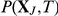 gives the probability of 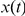 being in any of the states listed in 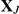 during the time 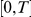, conditioned on the event of never leaving the inside region for any time 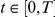. We can rewrite the probability as the conditional probability
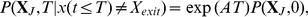
(13)where 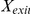 is the state to which the outside region 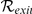 is aggregated. Remember that 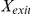 is an absorbing state. The probability of being inside the region 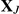 without ever leaving it during the interval 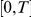 and the probability of visiting 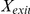 once should sum to one. Therefore
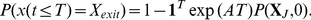
(14)Eq. 14 gives the probability of entering the region 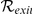 at least once within a time 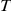. The boundary of the region that is aggregated into the absorbing state 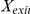, is chosen to include the states with a high number of ComK molecules. This indicates that the systems reaching the absorbing state corresponds to the cell being in a state of competence. Denoting 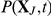 by 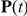 and 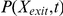 by 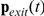 it can be seen that the probability of competence at time 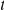, 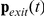, is given by
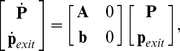
(15)where 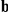 is chosen so that the columns of the state transition matrix add up exactly to zero.

### FSP for competence sensitivity

One advantage of having an analytical solution of the probability of competence is that we can use the solution to run a sensitivity analysis with respect to different model parameters. This makes it possible to shed light on the importance and roles that the different parts of the regulatory circuit play in reaching competence.

We start this section by introducing the equations we used to compute the sensitivity for the probability with respect to all the parameters. We then compare answers obtained by this method to estimates of sensitivities that we obtained using a finite difference method.

Recall that 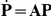 and suppose that we are interested in looking at the sensitivity of 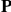 with respect to a parameter 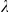, which could be any of the parameters presented in Table 1. The 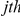 entry in 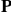 is given by 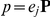, where 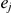, is an 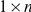 vector with 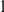 in the 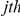 entry and zero everywhere else. We have from Eq. 9 that 
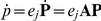
. Letting 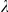 take values in the set of parameters 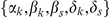, and using the fact that 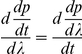, we get that 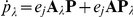 where 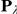 is defined to be 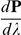. Similar equations hold for 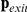. The sensitivity of the probability with which a cell enters in competence evolves according to the following dynamical system:
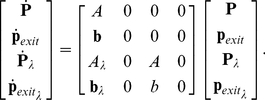
(16)Solving the above linear system, we obtain the sensitivity of the exit probability to all the parameters. We evaluate the solution at the nominal values given in Table 1. The results are reported in Table 2.

**Table 2:** Sensitivity of the probability of entering in competence.

System sensitivity					
	Sens 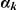	Sens 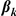	Sens 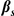	Sens 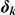	Sens 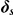
Method # 1	4.9931	8.4417	43.0166	−11.9632	−43.1321
Method # 2	4.9844	8.2370	42.3560	−11.9632	−43.0821

This table shows the sensitivity of the probability of entering in competence, as each of the indicated parameter varies, when the remaining parameters are set to their nominal values given in Table 1.

Using method 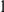, we solve the higher order system earlier introduced in the text. Using method 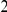, we used the solutions of the probability of entering in competence to numerically estimate the sensitivity of the system to various parameters.

For comparison, we calculated the same terms computed above by using a finite difference method. The sensitivity of the probability of entering competence with respect to the various parameters is calculated according to the formula 
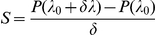
, where 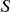 denotes the normalized sensitivity and 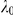 denotes the nominal value of the parameter of interest. In order to change study the sensitivity to each parameter, we update the value with small steps using the equation below
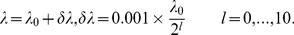
(17)


In summary, the sensitivity results presented in Table 2 are calculated using two different methods:

*Method #1:* We solve the double order system in Eq. 16. This results in more accurate answers but is more computationally expensive.

*Method #2:* We use the solutions for the original system describing the evolution of the probabilities of the states presented in Eq. 15 in addition to the numerical approximation method presented in Eq. 17 with 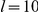. This method is less accurate than the first but is considerably faster to implement.

### FSP for expected duration of competence

We study here the time it takes for a cell to return from a state of competence to its original vegetative state. We use once again the analytical solution of the CME to conduct this analysis. We use a similar concept to the one explained earlier, with the difference that in this case, we aggregate into an absorbing state the region of the state space that corresponds to the vegetative state, indicating that the cell returned from competence. We also assume that the cell starts from a state of competence and that it is allowed to return from that state, i.e., competent states are no longer absorbing in this case. Starting from competence corresponds to starting from a pair (ComK,ComS) that falls anywhere in the region 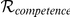. We assume that the cell can be at any state in 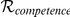 equally likely. This assumption translates to setting the initial probability vector 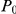 in a way that gives equal probability to all the states in 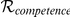. Return from competence is mapped to the region defined by 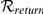. We set the initial probability vector to take the value 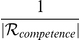 at the entries corresponding to the states in 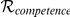 and zero everywhere else. Here 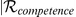 is the cardinality of the competence region in 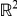. Having defined a region 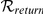 to be the region in the state space corresponding to return from competence, we aggregate all the states of return from competence into one absorbing state 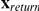. Hence, for the purpose of this calculation, once a trajectory ‘returns’ from competence, it cannot go back to it.

Having described the dynamics of the probability for return from competence in a similar manner to the description we had presented for the probability of entering in competence, we find the probability of returning from competence as a function of time by solving a set of differential equation just like we did earlier. We still need to deal with the infinite dimensions of the original model. For this purpose we add another absorbing state. This state is an aggregation of the region outside the finite state space that we consider, 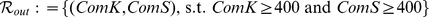, into a single state 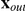. The finite state space is chosen so that the probability of reaching 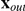 in the time interval of interest remains small. This small probability gives an upper bound on the approximation error due to the reduction of the infinite system into a finite one, as can be seen in the FSP algorithm [22]. Define 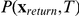 to be the probability of returning from competence within 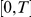. Denote by 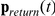, the probability 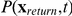 of returning from competence at time 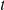, and by 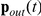, the probability 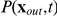 of exiting to the outside region at time 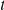.

The system becomes:
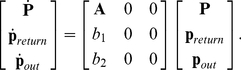
(18)


Now consider a partition of the interval 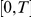 as follows:
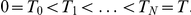
We can approximate the expected value of return time as follows:
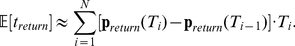
(19)


## Results/Discussion

**Figure 2:**
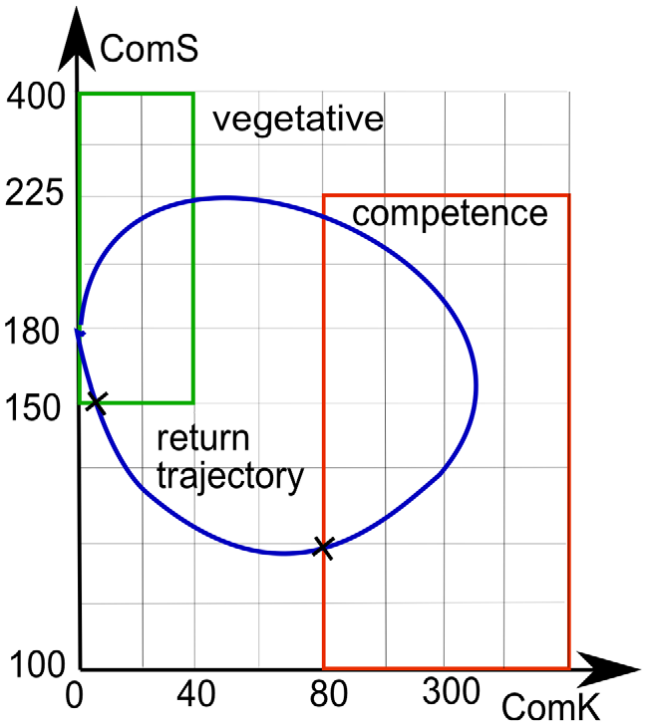
Return time for a sample trajectory. This figure shows how the regions were divided to compute the expected value of the time for which the cell remains in competence. The cross on the red rectangle is the start of the *return trajectory* of the cell from competence. The cross on the green rectangle is the end of the *return trajectory* to the vegetative state. We wish to calculate the expected time it takes for a cell to traverse such a trajectory.

We applied SSA to both the full model presented in Eq. 1–4, as well as to the reduced model presented in Eq. 7. We say that a cell entered in competence when the pair 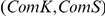 enter in the region 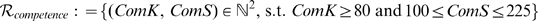. SSA simulations start from a number of molecules for 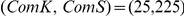 and all runs simulate 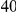 hours of molecular reactions. The initial number of molecules for ComK and ComS corresponds roughly to the mean steady state values of the reduced model. We are interested in studying the probability with which a cell enters in competence. For the return from competence analysis, we defined the region 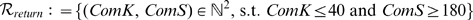. A cell return trajectory is the one it takes when going from 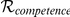 to 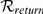 (see Fig. 2 for illustration). We should point out that the boundaries of the region may be selected regardless of their shape.

**Figure 3:**
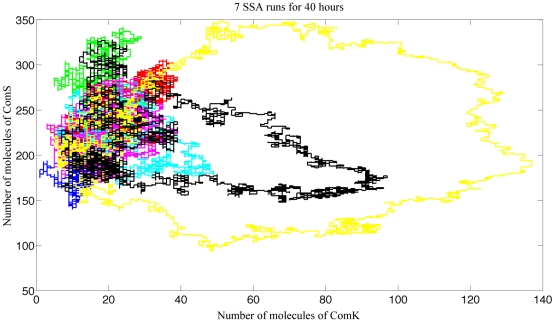
Seven SSA runs. This figure shows seven different SSA simulation runs of the competence regulatory genetic circuit. Each run is shown by a different color. Long trajectories correspond to high levels of ComK indicating that the cell has entered in a state of competence. This figure illustrates the stochastic nature of competence, by showing that starting from the same initial conditions, only two out out of seven cells enter in competence.

**Figure 4:**
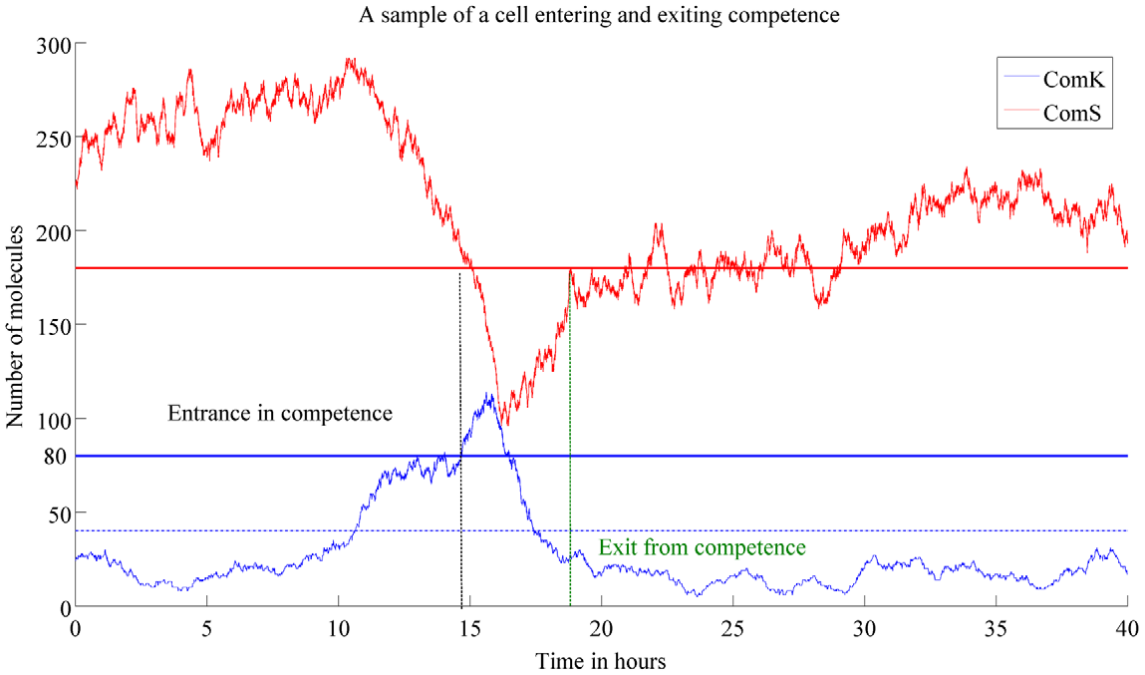
Single SSA run for 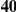 hours. This figure shows a single SSA run. The high level of ComK (shown in *blue*), as well as the negative correlations between ComK and ComS (shown in *red*) is a characteristic of competence.

In Fig. 3 we show seven different SSA runs, for 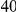 hours each. It can be seen that two of the runs behave differently from the remaining five runs. The long excursions seen in Fig. 3 correspond to a high number of ComK molecules, i.e., the state of competence. In Fig. 4 we show one SSA run where both ComK and ComS concentrations were plotted. Competence is clear in this case, and it is detected by both the high level of ComK and the negative correlation between ComK and ComS corresponding to the negative feedback from ComK to ComS when the number of molecules of ComK is high. In 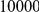 SSA runs, we found that the cell entered in competence 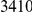 times, corresponding to an approximate probability 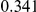. Using the **Chernoff inequality**, the accuracy in this case is described as 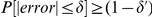, where 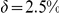 and 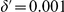 [23]. Using the bound on 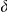 and Equation 19, we find an upper bound on the error in the calculation of 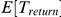.

Using the FSP based method as described in Eq. 15, we find that the probability of entering in competence at least once in 40 hours is 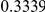, this probability is calculated with an error of no more than 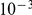.

### Sensitivity of entrance in competence

**Figure 5:**
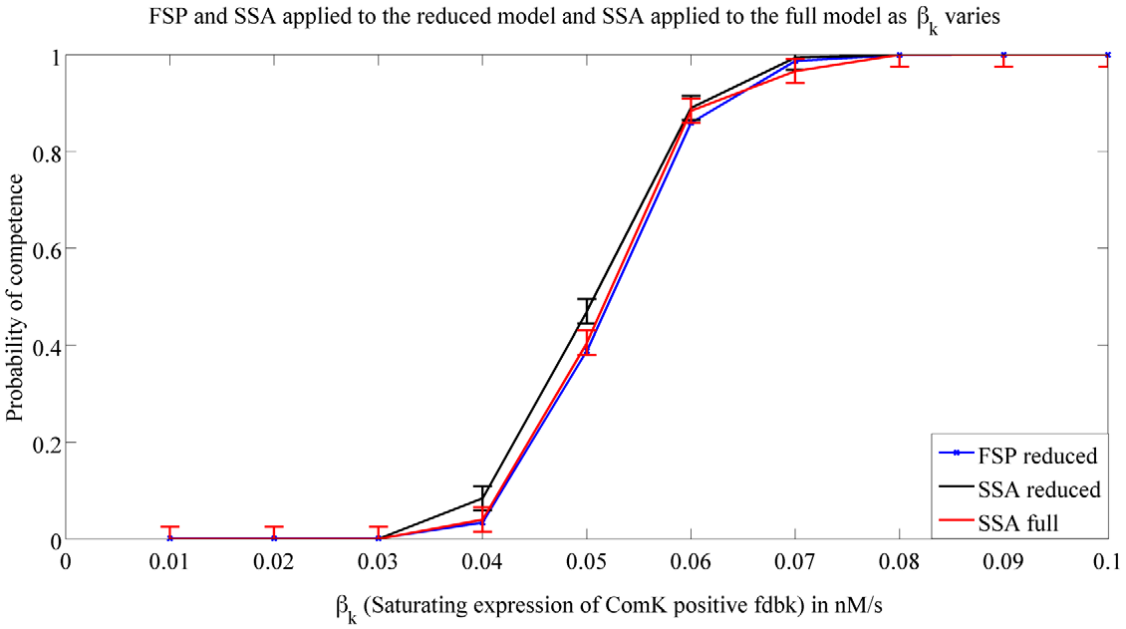
Probability of competence vs. 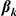. This figure shows the probability of entering in competence when 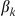 is varied. The three plots show simulations from the full model using SSA (*black*), the reduced model using FSP (*blue*) and the reduced model using SSA (*red*). SSA results were generated by averaging over 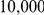 runs. For each data point, the error indicated by the errorbar is no larger than 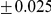 with a certainty no smaller than 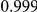. This is to be compared to an upper bound of 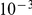 when using FSP.

**Figure 6:**
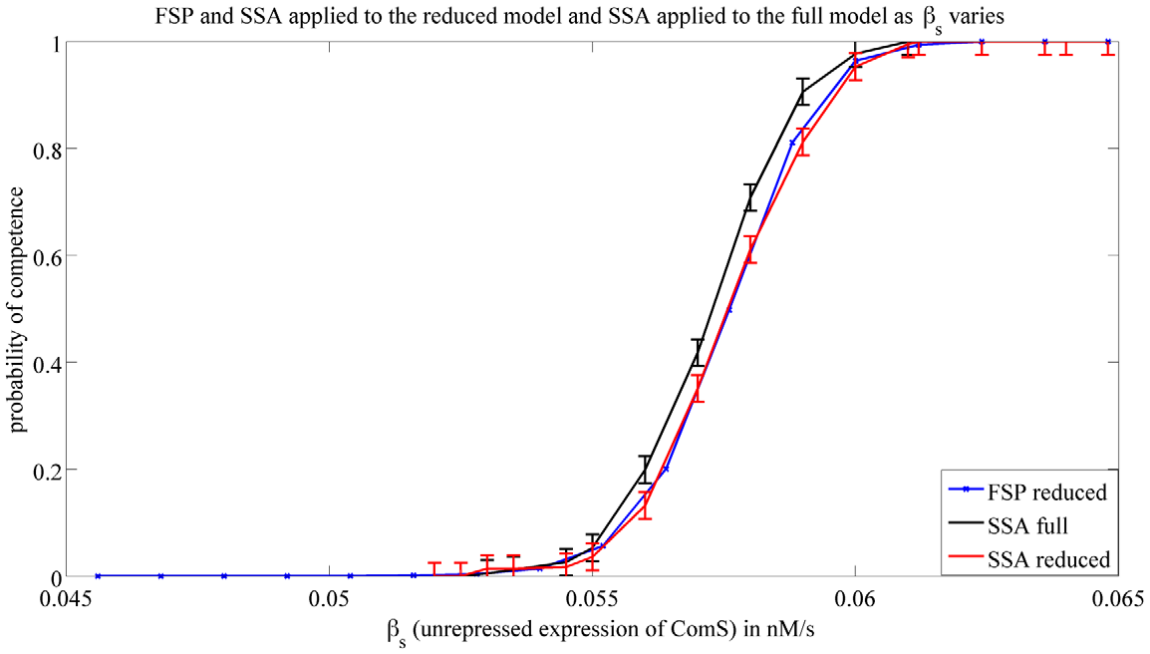
Probability of competence vs. 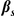. This figure shows the probability of entering in competence when 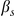 is varied. The three plots show simulations from the full model using SSA (*black*), the reduced model using FSP (*blue*) and the reduced model using SSA (*red*). SSA results were generated by averaging over 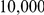 runs. For each data point, the error indicated by the errorbar is no larger than 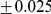 with a certainty no smaller than 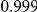. This is to be compared to an upper bound of 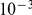 when using FSP.

Now that we presented the SSA, and FSP method, we first use the SSA algorithm to compare the reduced model in Eq. 5–6 to the full model in Eq. 1–4 both presented in [13]. In order to do this, we simulate both models using SSA and compare the probability of entering in competence as the parameters presented in Table 1 were changed. We show the results for the parameters 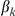, and 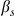 for demonstration purposes, but we note that the behavior of the full and reduced model were very close for all the parameters. We then compare the results given by the SSA and the FSP method, when applied to the reduced model. We show in Figs. 5 and 6, these results.

### Expected duration of competence

**Figure 7:**
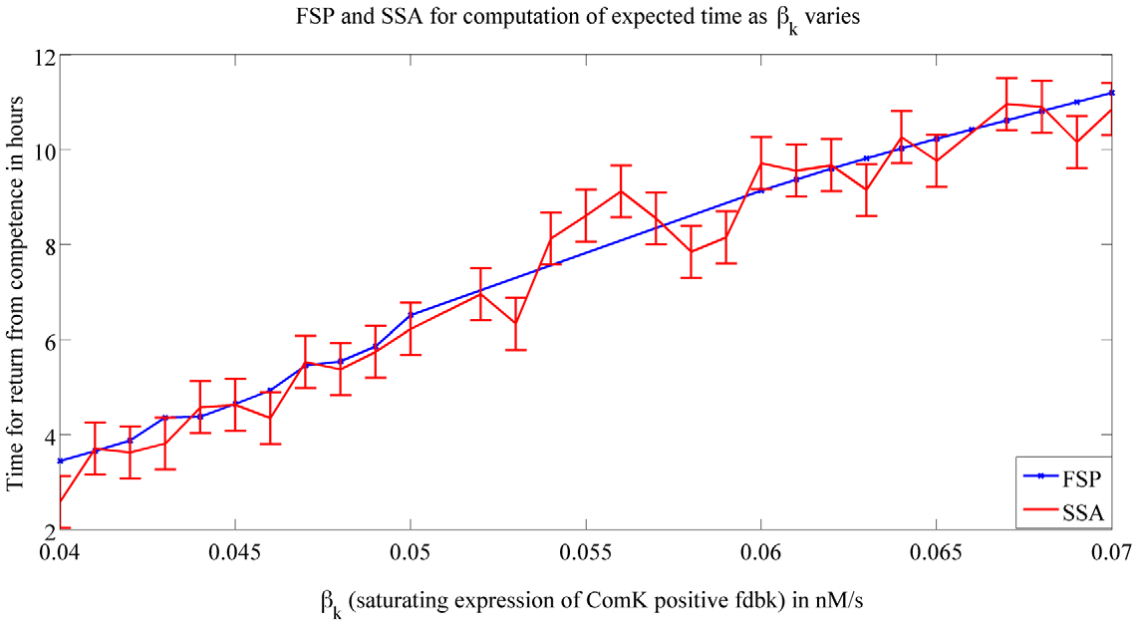
Expected time to return from competence vs. 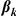. This figure shows the expected value it takes for a cell that started from competence to return to its vegetative state as 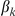 varies. The results are obtained using FSP (*blue line*) and SSA (*red line*). SSA results were generated by averaging over 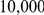 runs. For each data point, the error indicated by the errorbar is no larger than 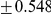 with a certainty no smaller than 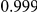. These results should be compared with the results obtained using FSP whose error has an upper bound of 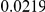.

We show next the insights our numerical methods allowed us to have about how the molecules involved in competence, affect the time a cell spends in this state. In Fig. 7 we show how changing the parameter 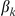 affects the time a cell stays in competence. This parameter corresponds to the saturation expression rate of the ComK positive feedback. The plot shows results obtained by both FSP and SSA. We can see that the plots exhibit similar behaviors, keeping in mind that such a calculation requires a lot more SSA simulations. In addition to giving more accurate results, the FSP approach allows us to combine multiple points from which we consider the cell as being in competence, while a different set of SSA simulations should be run for each different initial condition (starting number of molecules). Combining initial conditions is extremely useful in this case, since we care more about regions that the states go through than about specific points. It is not crucial to know the specific number of molecules of ComK or ComS when the cell is entering and returning from competence.

We saw earlier that increasing 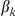 will increase the probability of cells entering competence. We now know that it will also keep the cell in competence for a longer time. Competence is an exhausting but occasionally necessary state for the cell. In this work we develop the CME accounting properly for the internal noise driving the competence switching dynamical system. The stochastic behavior of cell switching to competence has been studied in the literature. For example in their work, Süel et al. [13] account for the stochasticity by introducing an additive noise term to their model. The intensity of the noise and its distribution were parameters that are determined by the authors. In this work, we accounted for noise in its natural intrinsic form, eliminating therefore any controlled excitation of the excitable system.

We applied FSP to come up with an analytical solution, whereas other researchers always reverted to Monte-Carlo simulations, in their analysis. Finding an analytical solution made it possible for us to describe to a great extent the role of each of the molecules in driving cells into and out of competence. We discuss our results below.

We start by addressing the roles of the different expression and degradation rates in a cell entering competence. Fig. 5 shows that an increase in the saturating expression rate of ComK positive feedback 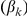 increases the probability of entering in competence. Fig. 7 also shows that it makes returning from competence slower. Although Figs. 5 and 6 show that ComK and ComS have similar roles in driving a cell into and back from competence, Table 2 suggests that changes in ComS affected by the values of the expression and degradation rates of ComS 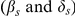 affect the probability of entering and staying in competence more than changes in ComK affected by the values of 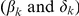. This leads to the expectation that the genetic circuits controlling ComS levels need to be much more sophisticated and complex than those regulating ComK in order to keep ComS concentration at specific values. Our normalized sensitivity analysis showed that increasing the basal expression rate 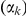 and the saturating expression rate of ComK 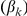 has an almost canceling effect to increasing the degradation rate of Comk 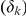 as far as the probability of entering in competence is concerned. It also showed that the expression and degradation rates of ComS 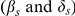, had a similar canceling effect. This means that each of these molecules plays a dual role. As it turned out, while the expression rate of Comk drives the cell in competence, its degradation rate brings it back to its vegetative state. Similarly, a high concentration of ComS drives the cell in competence by competing over free MecA with ComK molecules, leaving more ComK molecules free. On the other hand, a decrease in ComS is necessary to return from competence as we will see next. This is true because low levels of ComS allow free MecA molecules to bind to ComK decreasing therefore the level of ComK molecules. We saw as well that high levels of ComK and ComS drive the cell into competence with probability 1. This is in agreement with experimental results reported in [24], where Leisner et al use an approximate SDE model in which they account for noise by introducing an additive gaussian noise term, in contrast to our approach which uses CME directly.

**Figure 8:**
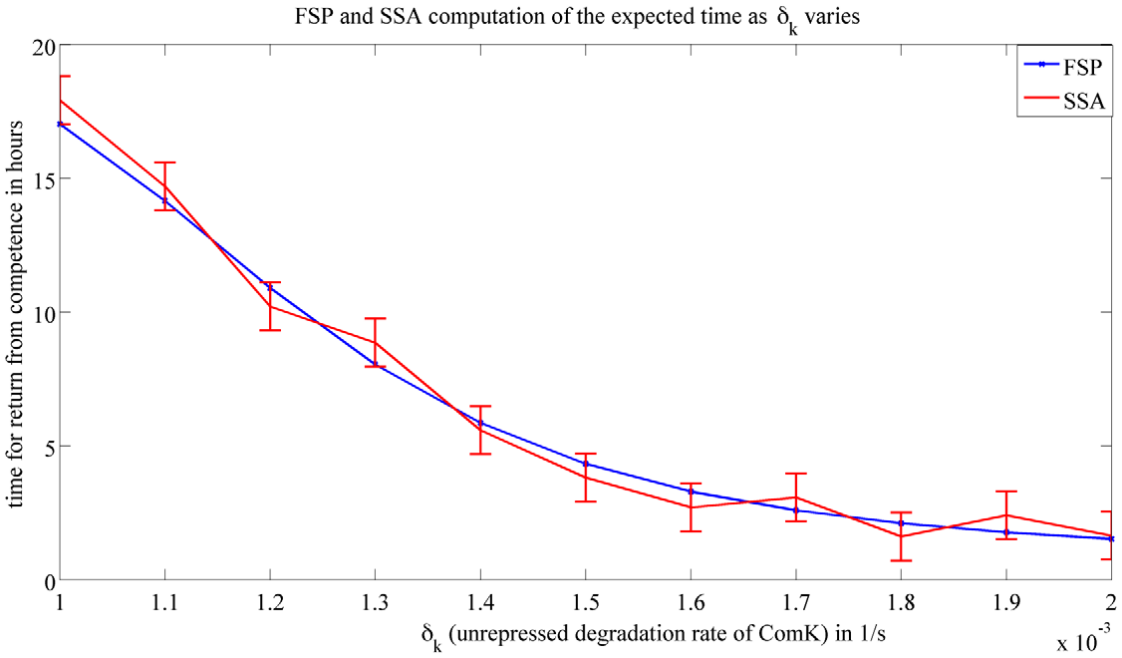
Expected time to return from competence vs. 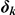. This figure shows the expected value it takes for a cell that started from competence to return to its vegetative state as 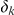 varies. The results are obtained using FSP (*blue line*) and SSA (*red line*). SSA results were generated by averaging over 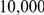 runs. For each data point, the error indicated by the errorbar is no larger than 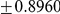 with a certainty no smaller than 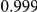. These results should be compared with the results obtained using FSP whose error has an upper bound of 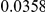.

We now study the roles of the different molecules in the return from competence. Figs. 7 and 8 suggest that the degradation rate 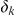 has a larger effect than 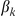 when it comes to the expected time for which a cell stays in competence. We found similar results for 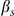 and 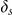. This implies that once a cell is in a state of competence, the degradation rate acts fast bringing it back to its vegetative state. The degradation rate is faster than the rate at which the free molecules try to keep the cell in competence. Fig. 8 suggests that increasing the value of 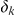 will decrease the time for which a cell stays in competence. We also know from Table 2 that an increase in 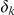 diminishes the probability with which *Bacillus subtilis* enters in competence. Our calculations also show that an increase in 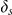 has a similar effect to an increase in 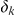 in the sense that they both decrease the probability with which a cell enters in competence and the expected time it takes for a cell to return form competence. Recall that 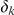 is the degradation rate of ComK, and 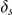 is the degradation rate of ComS. Also recall that whenever the number of ComS molecules is sufficiently small, more MecA molecules will be free to bind with ComK decreasing therefore the number of ComK molecules. Similarly, a higher ComK degradation rate, will lead to a decrease in the number of ComK molecules. A lower number of ComK molecules drive the cell back to its vegetative state and/or decreases its probability of entering in competence. This explains the similarity in the effect of 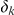 and 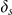 on the probability of entering competence and the expected return time.

### Conclusion

In this paper we developed a discrete stochastic model for competence in *Bacillus subtilis*. We performed simulations of the model using Monte Carlo based SSA and verified that the reduced order model gave a valid approximation of the full model. We then applied the recently developed FSP method to the reduced model and computed the probability of competence, where competence was defined in terms of a trajectory leaving a pre-defined region of the state space. Having the analytical solution, we were able to conduct a sensitivity analysis of the probability with which a cell enters in competence as the model parameters vary. We were also able to compute interesting terms such as the expected time it takes for a cell to return from competence.

This paper presented numerical methods that are applicable to many biological systems that exhibit a transient switching behavior. These methods were shown to be very useful in studying the genetic circuit regulating competence in a bacteria, and in answering questions about exact probabilities of stochastic events in this bistable biological behavior. They were also useful in studying sensitivities of these probabilities when expression rates, degradation rates, repression rates or activation rates of proteins were changed. Finally, the methods introduced in this paper showed how to calculate the expected time for return from transient states. Many other terms characterizing different transient physiological behaviors, such as the number of molecules that are most likely to enter in the transient states, and the return trajectories that are most likely to be taken can be computed using similar approaches to the one discussed here. Our approach should be easily extendible to analyze many biological system exhibiting a bistable switching behavior.
